# Ubiquitin E3 ligase activity in TRIpartite Motif (TRIM) family proteins

**DOI:** 10.1042/BST20260079

**Published:** 2026-09-16

**Authors:** Jane Dudley-Fraser, Katrin Rittinger

**Affiliations:** Molecular Structure of Cell Signalling Laboratory, The Francis Crick Institute, 1 Midland Road, London NW1 1AT, U.K.

**Keywords:** catalytic mechanisms, E3 ligase, structural biology, targeted protein degradation, TRIM, Ubiquitin

## Abstract

TRIpartite Motif (TRIM) family proteins are required for healthy development and homeostasis, while their dysregulation is observed in a wide range of diseases. Although diverse cellular roles are emerging for TRIMs, the majority function as ubiquitin E3 ligases by means of their RING (really interesting new gene) domain. Here, we delve into what is known about TRIM ubiquitin E3 ligase mechanisms, their ubiquitin chain specificities, and how they can be regulated through homo- and hetero-multimerisation and post-translational modifications.

## Introduction to the TRIM proteins

**Figure 1:**
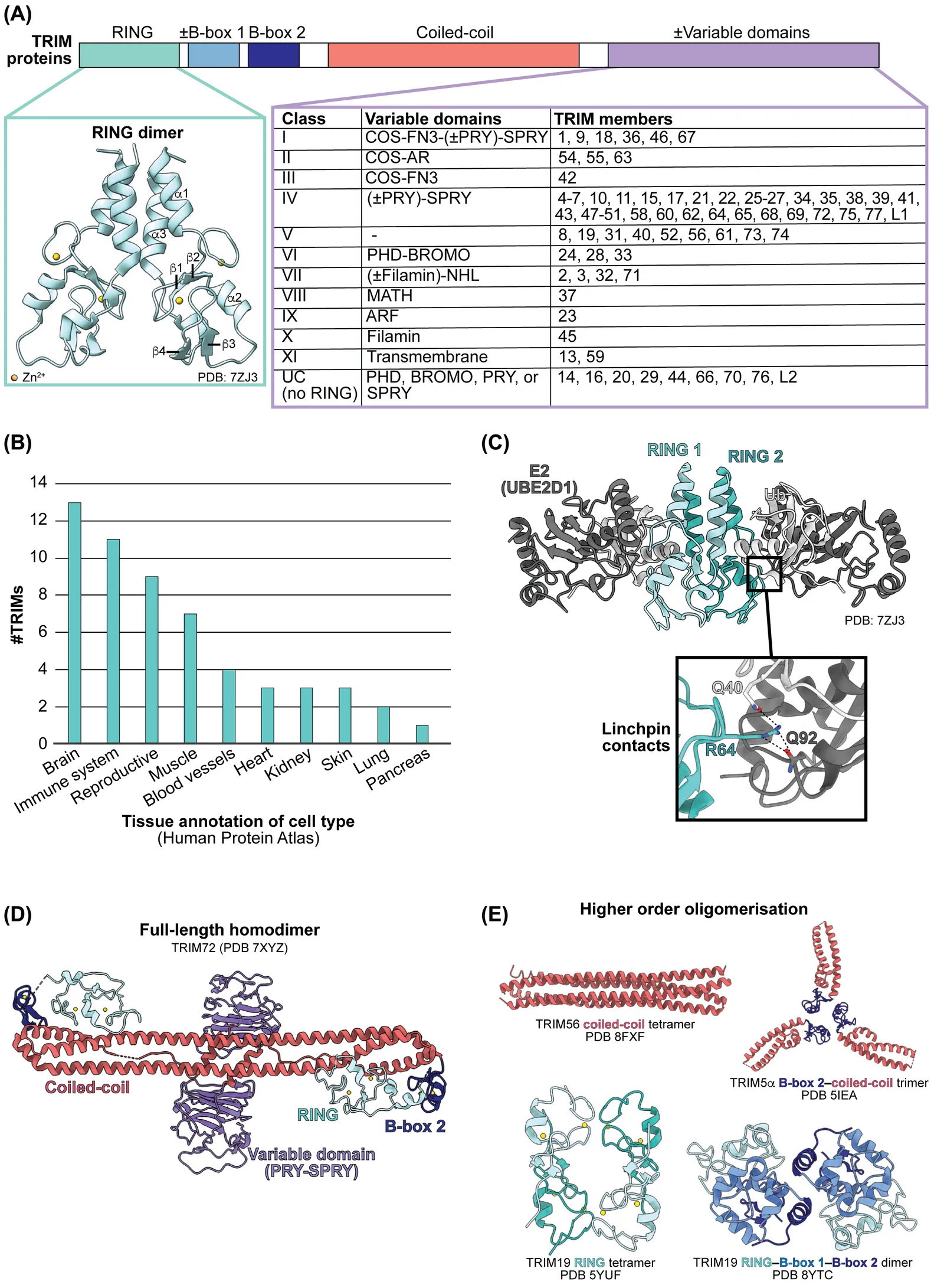
TRIM protein structure and expression (**A**) Schematic of TRIM domain organisation (top), alongside an archetypal TRIM RING (really interesting new gene E3 ligase) dimer structure (bottom left, adapted from TRIM2 PDB: 7ZJ3) and table of variable domain composition of TRIM subclasses (bottom right). (**B**) Cell-type tissue annotations of TRIM protein expression (Human Protein Atlas, proteinatlas.org). (**C**) TRIM2 RING dimers (turquoise) bound to UBE2D1∼Ub (dark and light grey, respectively) with an enhanced view of the R64 linchpin contacts. (**D**) Full-length TRIM72 homo-dimer structure solved by X-ray crystallography (PDB: 7XYZ). (**E**) Structures of TRIM oligomers available in the PDB (TRIM56 PDB: 8FXF; TRIM5α PDB: 5IEA; TRIM19 PDB: 5YUF; TRIM19 8YTC).

TRIpartite Motif (TRIM) proteins are defined by the presence of the eponymous tripartite RING–B-box–coiled-coil (RBCC) domain arrangement at their N-terminus. This is followed by variable C-terminal domains that sort them into 11 classes (Figure 1A). The 76 family members have been implicated in a plethora of biological functions in health and disease, most notably in brain function, cancer, and immunity [1,2]. This is mirrored in their reported basal tissue expression pattern, with the most frequently annotated cell types found in the brain and the immune system (Figure 1B). It should be noted, however, that the expression of many TRIMs increases in response to interferon stimulation [3–6]. To better understand the roles of TRIMs in pathophysiological contexts, which have already been covered extensively elsewhere, the present review will focus on what is known about how TRIM proteins function as ubiquitin E3 ligases, their regulation and specificity, and their potential for future drug discovery campaigns.

## RING-mediated ubiquitin transfer in the TRIM family

The presence of a conserved RING domain has led to the assumption that all TRIM proteins function as ubiquitin E3 ligases. Following the actions of E1 and E2 enzymes, E3 ligases catalyse the final step of ubiquitin transfer onto the substrate, resulting in substrates modified with ubiquitin monomers or poly-ubiquitin chains [7]. E3s provide specificity in the system by selectively binding targets for modification. E3 ligases come in three classes: RING, RING-between-RING (RBR), and homologous to the E6-AP carboxyl terminus E3 ligase (HECT), of which RBR and HECT function by binding ubiquitin covalently via a thioester bond [8]. In contrast, RING domains use multiple surfaces of weaker interactions to hold the E2∼Ub conjugate in a ‘closed’ conformation, which strains the thioester bond and primes it for discharge directly onto the substrate. While HECT and RBR E3 ligases dictate the ubiquitin chain linkage, in the case of RING domains this is dictated by the E2, as ‘the last guy’ that binds and thereby orientates the acceptor ubiquitin [9]. Depending on the linkage type and context, ubiquitin modification can result in many cellular outcomes, from signalling pathway modulation to transcriptional regulation to substrate degradation [7].

RING-mediated catalysis relies on multiple interaction interfaces, including a key RING β3–β4 loop that forms interactions with ubiquitin Q40 and the E2 α2–α3 region through a so-called ‘linchpin’ residue [10–13]. Intriguingly, all TRIM RING/E2∼Ub structures in the PDB are of TRIMs with an arginine at the linchpin position (Figure 1C). Interestingly, however, 30 of 66 (45%) RING-containing TRIM proteins possess a different residue at this position. The interactions that mediate the catalytic activities of these TRIMs remain to be uncovered. In the case of RNF38 RING, for example, mutagenesis studies show that alternative amino acids at the linchpin position can either reduce catalytic rate by two-thirds (e.g. lysine) or completely abrogate catalysis (e.g. tyrosine) [14].

**Figure 2:**
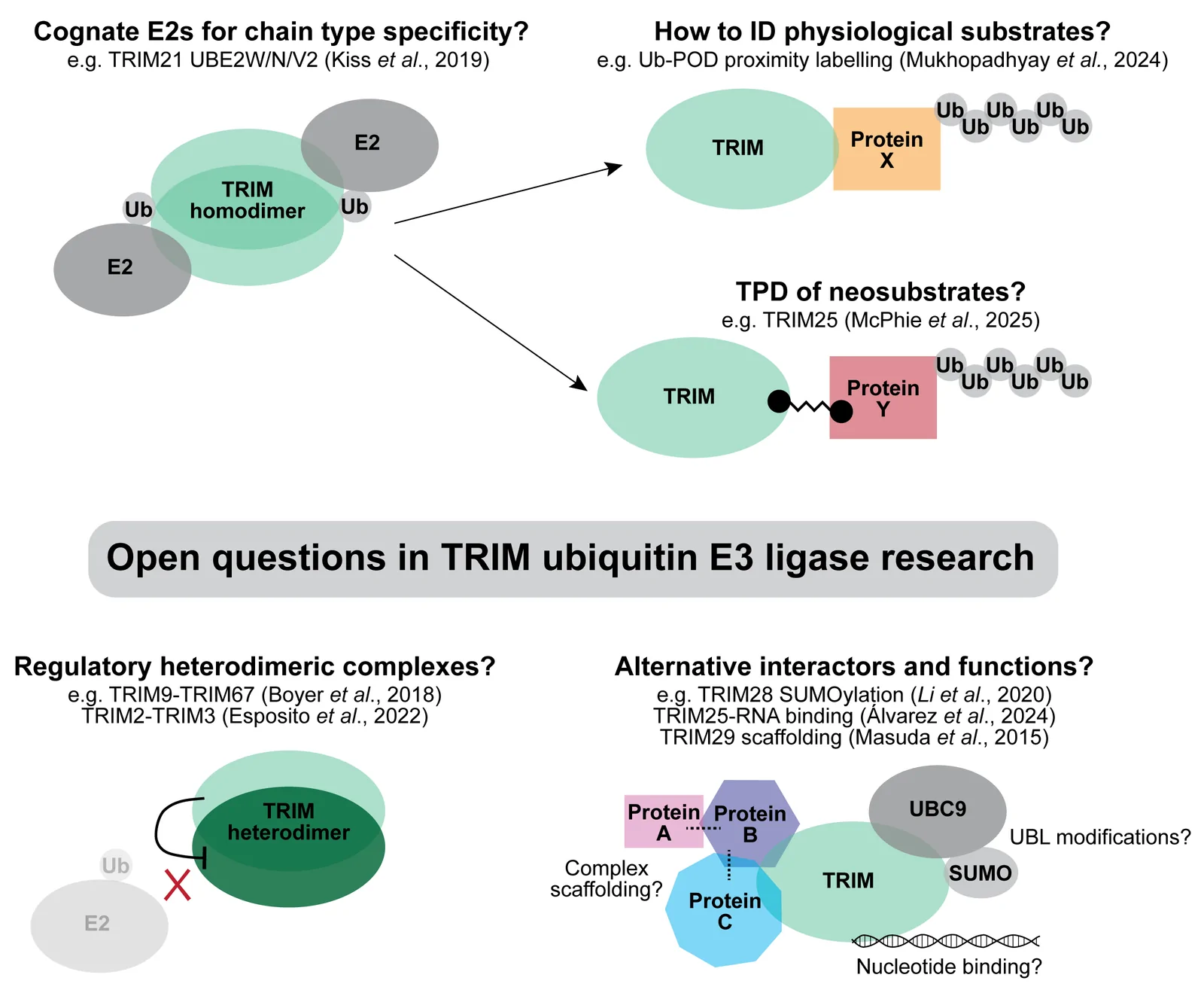
Areas for future TRIM research Illustrations representing open questions regarding TRIM ubiquitin E3 ligase activity.

The importance of the contribution of all RING/E2∼Ub interaction interfaces has been underscored by the identification of multiple inactive ‘pseudoligases’ in the TRIM family. Disruption of RING dimerisation, E2 binding sites, or the linchpin residue can ablate TRIM ubiquitination activity both *in vitro* and in cells [10,15,16]. The existence of ligase-dead RING-containing TRIMs is mirrored by the ‘unclassified’ TRIM class that lacks the RING domain (Figure 1A) [17,18]. The function of these TRIMs is currently largely unknown, although some appear to be able to exert negative regulation on active TRIMs (see below and Table 1). Alternatively, some pseudoligase TRIMs may play a role in supporting the catalysis of active RINGs, which is the case for another RING E3 complex, BARD1/BRCA1 [19]. These TRIMs may also carry out modification of substrates with ubiquitin-like proteins (UBLs), as has already been reported for several, including SUMOylation (e.g. TRIMs 11, 19, 28, and 32 [20–23]) and ISGylation (e.g. TRIM25 [24]). With significant improvements in biochemical and proteomic detection methods, there may yet be further examples of UBL modification activities waiting to be uncovered in the TRIM family (Figure 2). Then again, both unclassified and pseudoligase ubiquitin-deficient TRIMs harbour an array of domains that can mediate diverse protein–protein interactions (B-boxes, coiled-coil domains, and many of the C-terminal domains can all bind other proteins), and therefore they may act as catalytically inert scaffolds for larger complexes. For example, it has been shown that TRIM29 mediates assembly of DNA repair complex proteins in an E3 ligase-independent manner [25]. More work is needed to understand these hitherto unexpected non-catalytic functions of pseudoligases.

**Table 1:** TRIM proteins can form hetero-oligomers, some of which appear to be regulatory

TRIM–TRIM interactors	Interactors in the same class?	Impact of interaction on ubiquitination	Reference
TRIM2–TRIM3	Yes (Class VII)	TRIM3 represses TRIM2 activity	[10]
TRIM5α–TRIM21	Yes (Class IV)	TRIM5α ubiquitinated by TRIM21	[26]
TRIM9–TRIM67	Yes (Class I)	TRIM67 inhibits substrate ubiquitination by TRIM9	[27]
TRIM17–TRIM41	Yes (Class IV)	TRIM17 inhibits substrate ubiquitination by TRIM41	[28]
TRIM17–TRIM44	No (Class IV versus UC)	TRIM44 represses TRIM17 auto-ubiquitination	[29]
TRIM49–TRIM51	Yes (Class IV)	TRIM51 represses TRIM49 activity	[15]
TRIM1–TRIM18	Yes (Class I)	None reported	[30]
TRIM5α–TRIM6	Yes (Class IV)	None reported	[31]
TRIM5α–TRIM34	Yes (Class IV)	None reported	[31,32]
TRIM16–TRIMs 18, 19, 24	No (UC versus various classes)	None reported	[33]
TRIM17–TRIM39	Yes (Class IV)	None reported	[34]
TRIM24–TRIM28, 33	Yes (Class VI)	None reported	[35]
TRIM24–TRIM19	No (Class VI versus Class V)	None reported	[36]
TRIM24–TRIM27	No (Class VI versus Class IV)	None reported	[37]
TRIM38–TRIMs 5α, 27, 32, 35, 43, 45, 48, 49, 60, 70, 73, 74, L1	Some (Class IV versus various classes)	None reported	[38]
TRIMs 54, 55, 63	Yes (Class II)	None reported	[39]

## TRIM homo- and hetero-oligomerisation and post-translational modifications

Regulation of ubiquitin E3 ligase catalytic activity is critical to prevent target ubiquitination at inappropriate times or locations. While evidence of such regulatory mechanisms is beginning to emerge for some TRIMs, there appears to be variation between different family members and there is still much to learn.

TRIM proteins exhibit several levels of regulatory homo-multimerisation, as has been reviewed in detail elsewhere [40]. Firstly, the coiled-coil domain assembles into antiparallel homo-dimers (Figure 1D) [41,42]. Secondly, the RING can dimerise via α1 and α3, thereby generating surfaces spanning both protomers that co-operate in ubiquitin binding (Figure 1A). To satisfy both the coiled-coil- and RING-driven dimerisation requirements for catalysis, this would suggest TRIMs exist as homo-tetramers. Indeed, tetrameric tripartite RBCC complexes have been observed in solution by size-exclusion chromatography coupled with multi-angle light scattering (SEC-MALS) [10,43–45]. This model is further supported by tetrameric assemblies solved for isolated TRIM56 and TRIM75 coiled-coil constructs (e.g. TRIM56 coiled-coil Figure 1E) [46,47]. Importantly, such coiled-coil-mediated higher-order oligomerisation has been shown to be catalytically relevant. In a systematic family-wide analysis of TRIM proteins overexpressed in human cells, coiled-coil self-association was shown to drive clustering of some TRIMs into condensates and correlated with increased ubiquitination activity [38]. However, currently available TRIM structures in the PDB containing the RBCC are dimeric (TRIM28 RBCC, PDB: 6QAJ; TRIM72 full-length, PDB: 7XYZ) [48,49]. For TRIM28, this arrangement can be explained by coiled-coil-mediated dimerisation combined with the inability of its RING to dimerise and thereby generate tetramers [16]. In contrast, cryo-electron tomograph maps of TRIM72 suggest that RING dimerisation occurs transiently in a head-to-head fashion between oligomers in higher-order assemblies (rather than side-by-side via tetramerised coiled-coils), an arrangement that was not stable enough to be captured by X-ray crystallography [49].

Further oligomerisation can be promoted by the B-box domain. The B-box 2 of TRIM5α drives trimerisation, as demonstrated by SEC-MALS and X-ray crystallography using truncated engineered ‘mini-TRIM’ constructs (Figure 1E) [50,51]. Accordingly, in the presence of wild-type coiled-coil domains, full-length proteins assemble trimers of homo-dimers to form a hexagonal lattice [50,52]. Elegantly, trimerisation is important not only geometrically required for TRIM5α to make a neatly fitting net around its substrate (viral capsids) but also for its catalytic mechanism. While two of the RINGs form a dimer and bind E2∼Ub in the fashion described above, the third is positioned asymmetrically and acts as a recipient for autoubiquitination [53]. The B-box 2 of TRIM19 (PML), on the other hand, appears to disrupt the tendency of the RING to tetramerise into a ‘taurus’ assembly (PDB: 5YUF), with the RING–B-box 1–B-box 2 structure instead adopting an atypical dimer conformation mediated by α1 of B-box 2 (PDB: 8YTC, Figure 1E) [54,55]. Biologically, TRIM19 B-box 2 is required for its role in nuclear bodies (NB), and disrupting its dimerisation ablates NB formation, suggestive of a key conformational constraint [56]. As most molecular analyses have focussed on isolated RING domains, however, it remains to be seen whether the B-box domains of any other TRIMs may contribute to higher-order oligomerisation.

B-box 2 of TRIM21 regulates RING ubiquitin E3 ligase activity by a different means. In this case, the B-box competes with the E2 binding site on the RING domain, which is then released when Ser80 in the RING domain is phosphorylated by IKKβ or TBK1 [57]. TRIM21 ubiquitination activity can also be blocked by N-terminal acetylation, while its ISGylation can enhance it [58,59]. Alternatively, phosphorylation of TRIM23 promotes its ubiquitination activity, as well as its proposed role as a GTPase [60]. It is interesting to consider how other TRIMs may be similarly regulated by post-translational modification following specific cellular cues, which may have been missed in studies thus far [15,38].

A post-translational modification of TRIMs that has been frequently observed, however, is auto-ubiquitination. TRIM auto-ubiquitination is well-documented both *in vitro* and in cells (e.g. TRIM2, TRIM5α, TRIM21, TRIM23, TRIM32, and TRIM56) [10,15,38,61–63]. RING deletion or mutation at key residues (e.g. linchpin or zinc co-ordinating residues) can not only affect the ability of a TRIM to ubiquitinate substrates, but can also increase its own stability, suggesting TRIMs may regulate their own protein levels in cells by auto-ubiquitination when substrates are unavailable [10,38,63,64]. For example, preventing auto-ubiquitination stabilises TRIM21 and so increases its substrate targeting window [58].

Another reported regulatory mechanism of TRIM activity is hetero-oligomerisation. Many TRIM family members have been observed to interact with one another or co-localise in cultured cell lines, some of which appear to be regulatory (Table 1). For example, the binding of one TRIM to another can inhibit its ubiquitin E3 ligase activity (e.g. TRIM51 represses TRIM49 activity [15]). This may represent a possible function for catalytically inactive pseudoligase TRIMs.

These findings raise two main questions. Firstly, how often do interactions result in activity regulation? Many of the studies reporting interactions have not probed whether the interaction impacts ubiquitin E3 ligase activity. Secondly, are all hetero-oligomers physiologically relevant? While it is tempting to suggest interacting pairs of TRIMs represent a common regulatory mechanism, this is at odds with the differential tissue expression distributions of some of the reported TRIM pairs (e.g. TRIM2 and TRIM3 localise to different regions of the brain; TRIM1 and TRIM18 expression patterns partially overlap during development but are distinct in adults [10,65]). Notably, many of these reported hetero-oligomers form between closely related TRIMs from the same class. It is possible that these closely related TRIMs may be the result of gene duplication and then evolved distinct functions in different tissues, with the capability to hetero-dimerise merely a relic of their shared history. Further work is required to understand the relevance of these interactions *in vivo* (Figure 2).

## TRIM ubiquitination substrates and specificity

While TRIM ubiquitin E3 ligase activity has been shown to require certain structural features, such as multimerisation, there are a limited number of studies examining how substrate, ubiquitin chain, or spatiotemporal specificity arises (Figure 2).

Substrates have been proposed for many TRIMs, which are too numerous and varied to cover in full here. Of these, the most complete mechanistic data are available for TRIM5α and TRIM21. Interestingly, these studies show that these TRIMs are their own ubiquitination substrates, with their C-terminal PRY/SPRY domains acting as anchors to bring viral components along for degradation without ubiquitinating them directly [66,67]. Elsewhere, identifying ubiquitin E3 ligase substrates is still not a trivial endeavour, despite advances in ubiquitin-specific proximity labelling techniques [68–70]. Moreover, the validation of *bona fide* substrates using recombinant proteins remains scarce in the field, with many studies correlating *in cellulo* substrate ubiquitination status with co-immunoprecipitation with the TRIM of interest. *In vitro* validation is critical, given the indirect nature of such correlations and the difficulty in capturing E3–substrate interactions, which are necessarily transient for catalytic throughput. Therefore, more work is required to comprehensively identify and characterise TRIM ubiquitination substrates under physiological conditions and validate them *in vitro*.

Once substrates have been identified, another significant challenge is to understand how TRIM activity is triggered in a context-specific manner. One such mechanism has been found to be activation upon binding to a biological sensor. In the case of TRIM25, binding to viral RNA appears to enhance its ubiquitination activity, thereby offering a mechanism by which it only switches on under appropriate conditions to ubiquitinate innate immune regulators such as RIG-I and ZAP [71–73]. Interestingly, many other TRIMs have also been implicated in viral restriction, and several have been found to bind RNA (e.g. class VII TRIMs 2, 3, 32, 71; TRIM56), but thus far its impact on their E3 ligase activity remains to be examined [74,75].

Another area that warrants further exploration is the question of ubiquitin chain linkage specificity, which in the case of RING domains equates to E2 binding specificity [9]. This is a critical point given the distinct cellular outcomes brought about by different linkages, such as proteasomal targeting of K48-linked chains [7]. Particular ubiquitin chain linkage types, as determined using ubiquitin lysine point mutants or western blotting with linkage-specific antibodies, are often reported in TRIM–substrate relationships, hinting at functionality with a particular E2 enzyme. However, a limited number of studies have comprehensively examined the E2 preferences of TRIMs. A yeast-two-hybrid screen by Napolitano et al. found relatively few TRIM:E2 interactions, although the authors note that due to the transient nature of such interactions, they may not all be captured by this methodology [76]. In the cases of both TRIM5α and TRIM21, while they can function with other E2s (e.g. UBE2D family members), their biological functions require the sequential action of UBE2W and UBE2N/V2 enzymes to initiate and then extend K63-linked chains of auto-ubiquitination, respectively [11,53,63,77]. Elsewhere, TRIM17 has been shown to preferential activity with UBE2N/V1, while TRIM2 and TRIM39 are less selective and can function with UBE2Ds, UBE2Es, and UBE2N/V1 [10,34]. It will be interesting to understand whether—and structurally how—other TRIMs exhibit a preference for particular E2s to inform chain linkage type and, therefore, physiological outcomes.

## TRIM small molecule and TPD ‘druggability’

In light of their importance across many areas of biology, TRIMs have attracted attention not only as drug targets but also as potential components of targeted protein degrader (TPD) modalities [78].

TRIM small molecule inhibition may target either the RING domain (to block ubiquitin E3 ligase activity) or the C-terminal variable domains (to prevent substrate binding or induce TRIM TPD, e.g. the TRIM24 bromodomain is the target for a VHL-driven protein-targeting chimera (PROTAC)) [79]. The challenge lies in the lack of a single ‘catalytic’ residue to target and the co-operative, shallow nature of the TRIMs' interaction interfaces. Therefore, it will be most practical to utilise covalent ligands that overcome weak affinity and aid detection of engagement [80]. Small molecule ligands binding the SPRY domains of TRIMs 7 and 58 have been reported, as well as a covalent ligand for the SPRY domain of TRIM25, demonstrating a potential for druggability [81–83].

Meanwhile, TPD has the advantages of combining target engagement flexibility with favourable pharmacology. Heterobifunctional and molecular glue development is a rapidly growing and maturing field, with the first PROTAC receiving FDA approval in May 2026 [84]. TRIMs offer a way to diversify current largely Cullin RING-based modalities and, excitingly, targeted degraders employing TRIM21 (‘TRIMTACs’) are already being explored [85–87].

However, in order to capitalise on these approaches, it is first vital to glean more comprehensive data in four key areas (Figure 2):

1. Which TRIMs are catalytically capable of ubiquitination *in vivo* and do any PTMs or interactors regulate them? Many studies of TRIM ubiquitination activity have been conducted *in vitro*, potentially missing any cellular activators or repressors.
2. Which TRIMs are expressed in the cell type/tissue of interest? Are they tissue-specific or will off-target tissues also be affected? More work is required to determine the roles of TRIM proteins on an organismal level.
3. What are their substrates in physiological contexts?
4. Which E2s do they function with and what type of ubiquitin chains are they capable of forming?

These are fascinating but challenging questions that will inform not only our fundamental understanding of TRIM ubiquitin E3 ligase activity but also the means by which they can be manipulated in the treatment of diverse diseases. With appropriate chemical tools and proteomic analyses now coming to the fore, TRIM therapeutic development is a compelling area for future research.

## Perspectives

- TRIM protein RING-mediated ubiquitin E3 ligase activity requires varying degrees of RING self-association in order to engage the E2∼Ub conjugate in the ‘closed’ conformation for ubiquitin discharge onto substrates.
- While some substrates, regulatory mechanisms, and specificities have been identified, the majority of TRIMs lack such mechanistic understanding.
- Advances in ubiquitin-specific proximity labelling techniques mean physiologically relevant TRIM E3 ligase substrates can now be identified more readily. *In vitro* validation of these substrates, analysis of E2 and ubiquitin chain specificity, and elucidation of spatiotemporal regulatory mechanisms will set the scene for drug discovery efforts.

## Open Access

Open access for this article was enabled by the participation of the Francis Crick Institute in an all-inclusive Read & Publish agreement with Portland Press and the Biochemical Society under an agreement with JISC.

## Abbreviations

HECT: homologous to the E6-AP carboxyl terminus E3 ligase
ISGylation: modification with interferon-stimulated gene 15 (ISG15)
NB: nuclear bodies
PROTAC: protein-targeting chimera
RBCC: RING–B-box–coiled-coil motif
RBR: RING-between-RING E3 ligase
RING: really interesting new gene E3 ligase
SEC-MALS: size-exclusion chromatography coupled with multi-angle light scattering
SUMOylation: modification with small ubiquitin-like modifier (SUMO)
TPD: targeted protein degradation
TRIM: TRIpartite Motif
UBLs: ubiquitin-like proteins
